# An integrated stress-adaptation framework for understanding mental health in developmental coordination disorder and comorbid neurodevelopmental conditions (ISAF-DCD)

**DOI:** 10.3389/fpsyg.2026.1965095

**Published:** 2026-10-29

**Authors:** Nadja Schott

**Affiliations:** Department for Sport Psychology and Human Movement Science, Institute for Sport and Movement Science, University of Stuttgart, Stuttgart, Germany

**Keywords:** developmental coordination disorder, environmental stress hypothesis, lifespan adaptation, neurodevelopmental disorders, psychosocial outcomes, resilience, stress framework

## Abstract

Developmental Coordination Disorder (DCD) is associated with elevated rates of anxiety and depression, as well as reduced quality of life, yet the mechanisms linking motor difficulties to these outcomes remain incompletely specified. This article presents and critically evaluates the Integrated Stress-Adaptation Framework for DCD (ISAF-DCD), which synthesizes empirical findings on DCD with established stress theories, including the Environmental Stress Hypothesis, the transactional model of stress, and the Conservation of Resources model. Although the framework is presented with reference to comorbid neurodevelopmental conditions broadly, its evidential basis and worked examples concentrate specifically on attention-deficit/hyperactivity disorder (ADHD) and autism spectrum disorder (ASD); generalization to other neurodevelopmental conditions is plausible but has not been evaluated here. ISAF-DCD proposes that motor coordination difficulties influence mental, cognitive, and physical health through interacting environmental demands, primary and secondary stressors, appraisal and response processes, and personal and social factors. The framework is bidirectional and developmentally sensitive, allowing outcomes to feed back into subsequent stress and adaptation processes across the lifespan. I examine the model’s conceptual coherence, empirical support, and potential clinical utility, arguing that ISAF-DCD offers a theoretically grounded heuristic for organizing a fragmented literature while remaining provisional. Its proposed contributions include clarifying the multiple roles of resilience and social support, refining the classification of environmental demands, incorporating neurodevelopmental comorbidity as a cross-cutting factor, and representing reciprocal relationships between stress and motor functioning. To improve falsifiability, I specify the temporal and functional roles of key dual-role constructs and derive five core, empirically distinguishable propositions that differentiate ISAF-DCD from an expanded Environmental Stress Hypothesis. Direct empirical support for some components, particularly the proposed corticolimbic mechanisms, remains limited. ISAF-DCD should therefore be treated as a hypothesis-generating framework, focused primarily on ADHD and ASD as comorbid conditions and on mental health as its central but not exclusive outcome domain, that requires rigorous longitudinal and intervention-based evaluation.

## 1 Introduction to developmental coordination disorder

Developmental Coordination Disorder (DCD) is among the most prevalent neurodevelopmental conditions, affecting approximately 7% of boys and 4% of girls. It is characterized by marked impairment in acquiring and executing coordinated motor skills that significantly interferes with activities of daily living (ADL) and academic performance (Blank et al., 2019; Li et al., 2024). DSM-5-TR criteria specify that motor difficulties must manifest early in development, cannot be better explained by intellectual disability or a neurological condition, and must cause functional impairment (American Psychiatric Association, 2022). Despite this relatively clear nosology, DCD remains substantially under-recognized and underdiagnosed in clinical practice, contributing to unnecessary stress for affected individuals and their families (Klein et al., 2024; Meachon et al., 2024; Murray et al., 2025).

Common characteristics of DCD include slow, inefficient, and poorly coordinated fine- and gross-motor movements (Schott, 2019). However, the clinical presentation is heterogeneous. Cluster-analytic studies have identified patterns of motor, cognitive, and visuomotor performance that may distinguish subgroups of children with DCD (Gras et al., 2023; Lust et al., 2022). Some children show generalized difficulties across fine-motor, gross-motor, and balance domains, whereas others show more circumscribed difficulties in manual dexterity, balance, motor planning, or visuospatial processing (Lust et al., 2022). A recent systematic review found no consistent evidence for stable, discrete DCD subtypes and argued that DCD may be better conceptualized as a spectrum of severity and pervasiveness (Verbecque et al., 2026). Taken together, these findings suggest that individuals with DCD vary along overlapping dimensions and that proposed subtypes should be regarded as provisional descriptions rather than established categories. Cognitive profiles are similarly heterogeneous, with executive-function difficulties involving planning, working memory, and inhibition varying in nature and severity (Van Dyck et al., 2022).

DCD frequently co-occurs with other neurodevelopmental conditions, particularly attention-deficit/hyperactivity disorder (ADHD) and autism spectrum disorder (ASD). Estimates of co-occurring DCD and ADHD range from 30 to 50%, and twin studies indicate a substantial shared genetic component between DCD and ADHD (Meachon et al., 2025; Micai et al., 2023; Mountford et al., 2021). Fine-motor difficulties may be particularly associated with the inattentive presentation of ADHD. Motor-coordination difficulties are also common in ASD, although prevalence estimates vary by screening instrument, diagnostic threshold, and sample characteristics. Notably, co-occurring ADHD and DCD may represent a more severe and neurocognitively distinct presentation rather than the simple addition of two independent conditions (Pranjić et al., 2023).

Contrary to earlier assumptions that children outgrow their motor difficulties, DCD often persists into adolescence and adulthood. Longitudinal studies indicate that coordination difficulties may continue to affect daily functioning, education, employment, and social participation across the lifespan (Järvinen et al., 2024; Sauer et al., 2026). Evidence of elevated anxiety and depression, together with reduced quality of life (QoL), has therefore encouraged theoretical work on pathways linking motor difficulties to mental health outcomes (Fortnum et al., 2026; Michelini et al., 2024). Against this backdrop, ISAF-DCD is introduced as an attempt to integrate motor, psychosocial, and stress-related processes into a single developmental framework.

### 1.1 Rationale for the integrated framework (ISAF-DCD)

Existing accounts of the relationship between DCD and mental health have often focused on specific mechanisms, most notably the Environmental Stress Hypothesis (ESH). The ESH conceptualizes motor impairment as a primary stressor that may lead to secondary psychosocial stressors and, ultimately, internalizing symptoms (Cairney et al., 2013). Although this account has been productive, it was not designed to specify the cognitive appraisal processes that intervene between exposure and response, incorporate neurobiological processes, or differentiate among multiple health outcomes across the lifespan. General stress theories, including the transactional model of stress (Lazarus and Folkman, 1984) and diathesis-stress models (Zuckerman, 1999), address some of these processes but were not developed with DCD’s motor, social, and developmental features in mind.

ISAF-DCD is proposed as a synthesis of existing models, not a replacement. It retains the ESH distinction between primary and secondary stressors while adding cognitive appraisal, potential neurobiological correlates, a broader set of mediating and moderating factors, and explicit feedback loops. The framework is intended to organize multiple plausible pathways from motor difficulties to mental, cognitive, physical, and behavioral outcomes rather than to assert a single causal mechanism. The central premise is that the mental-health consequences of DCD are unlikely to be explained adequately by motor impairment alone or by any single stress theory.

The literature underlying ISAF-DCD was identified through a narrative, non-systematic approach that combined recent systematic reviews and meta-analyses on DCD, ADHD, and ASD with targeted searches on stress theory, corticolimbic function, and developmental psychopathology. This approach is appropriate for a Hypothesis and Theory article but means that evidence selection may be subject to the author’s judgment rather than to the pre-specified inclusion criteria of a systematic review; this limitation is revisited in the Conclusion and partially addressed by the evidence map in Section 2.11.

### 1.2 Scope, aims, and the status of ISAF-DCD as a framework

Two scope decisions need clarification before presenting the framework. First, although the title refers broadly to comorbid neurodevelopmental conditions, the framework and supporting evidence focus specifically on ADHD and ASD, which are the neurodevelopmental conditions most consistently reported to co-occur with DCD and for which mental-health-relevant evidence is most available (Meachon et al., 2025; Micai et al., 2023; Mountford et al., 2021). Generalization to other neurodevelopmental conditions is plausible but has not been evaluated here and should not be assumed. Second, although the title emphasizes mental health, ISAF-DCD explicitly incorporates brain and cognitive health, physical health, and health-related behavior as outcome domains. Mental health is the primary and best-evidenced endpoint. At the same time, the other domains are included because they plausibly interact with mental-health outcomes through shared mediators (e.g., physical activity, executive function), not because ISAF-DCD is intended as a fully general framework of multidimensional adaptation.

ISAF-DCD is best understood as serving three related but distinguishable functions: (1) an organizing framework that integrates the fragmented DCD-stress literature under a common structure; (2) a heuristic for generating developmental research questions; and (3), to a more limited and currently aspirational extent, a source of falsifiable, empirically distinguishable predictions. The synthesis in Section 2 well supports the first two functions. This article substantially strengthens the third function through the construct-role table (Section 2.8) and the core falsifiable propositions (Section 2.10), but ISAF-DCD is not yet a fully predictive theory, and readers should weigh its claims accordingly. Section 3.3 discusses this trade-off between comprehensiveness and predictive constraint in more detail.

## 2 Critical analysis of framework components

For each component of ISAF-DCD, this paper considers four questions: (1) Is its inclusion supported by empirical research on DCD? (2) Is it appropriately classified as a stressor, mediator, moderator, or outcome? (3) Are the proposed directional relationships empirically justified?, and (4) What gaps limit evaluation of the component?

### 2.1 Environmental demands

ISAF-DCD distinguishes environmental demands from the person-level characteristics and broader cross-cutting factors. Here, environmental demands are restricted to situational and contextual conditions that are, at least in principle, external to the individual: novelty, predictability, uncertainty, ambiguity, task complexity, dual-task requirements, and access to intervention and support. Age, sex, gender, goals, and values are instead treated as person-level characteristics that interact with these demands.

#### 2.1.1 Situational factors

Children with DCD may experience particular difficulty in novel or unpredictable environments, possibly reflecting differences in predictive processing and internal models. Dual-task paradigms also commonly show larger performance decrements in children with DCD than in typically developing peers (Subara-Zukic et al., 2022; Van Dyck et al., 2022). These findings support including task characteristics as potential environmental demands, although their specific contribution to mental health outcomes remains less well established. Access to therapy, specialized schooling, and diagnostic services should not be treated merely as covariates that alter the strength of existing associations. They are contextual conditions that can change the demands placed on an individual. For example, a child exposed to high motor demands but limited access to intervention may experience a different stress profile from a child with comparable motor difficulties who receives appropriate support (De Roubaix et al., 2025).

Access to intervention and support could alternatively be classified as a contextual resource, moderator, or form of intervention exposure rather than a demand, and this classification is a genuine judgment call. ISAF-DCD retains it under environmental demands for two reasons. First, unlike the mediators and moderators discussed in Section 2.9, access to intervention is not a within-person capacity or response tendency; it is a feature of the child’s surrounding environment, consistent with how “demand” is used here to mean any external, situational condition the individual must contend with, not only conditions that are burdensome (Meachon et al., 2024). Second, and more importantly, access to intervention functions dually: it is simultaneously a structural feature of the environment and, once present, a resource that can buffer the impact of other demands, resembling the dual-role constructs marked. Its placement in the environmental-demands category should therefore be read as marking its origin (an externally determined condition) rather than a claim that it never functions as a moderator; its moderating function is addressed explicitly where physical activity and family support are discussed as mediators and moderators (Sections 2.4 and 2.9).

#### 2.1.2 Person-level and developmental factors

Age and developmental stage influence the types of motor challenges individuals encounter and the social meaning of those challenges (Cairney, 2018). Because developmental stage may moderate the strength of the pathway from motor difficulty to psychosocial outcomes, with the same level of impairment may have different consequences at ages 5 and 15, it is better represented as a cross-cutting factor in Figure 1 rather than as a static environmental demand or a fixed individual characteristic. Sex and gender differences in DCD presentation and mental-health outcomes are increasingly recognized, although the empirical base remains limited. Some findings suggest that girls and women with DCD may be particularly vulnerable to anxiety during adolescence (Erskine et al., 2024; Harris et al., 2025). Future work should distinguish sex-related biological influences from gendered expectations, social experiences, and differential access to support. Consistent with the taxonomy, age, sex, and gender are treated as person-level characteristics that condition how environmental demands are experienced, rather than as demands themselves. Goals and values may similarly function as person-level moderators of appraisal. Achievement orientation, social goals, and the personal importance attached to motor performance may influence whether a motor challenge is appraised as threatening, manageable, or relatively unimportant (Dahl et al., 2024; Pelletier-Baldelli et al., 2022). Direct research in DCD is needed to test whether goals and values are better modeled as stable moderators or as inputs to the appraisal process itself (see Section 2.8).

**Figure 1:**
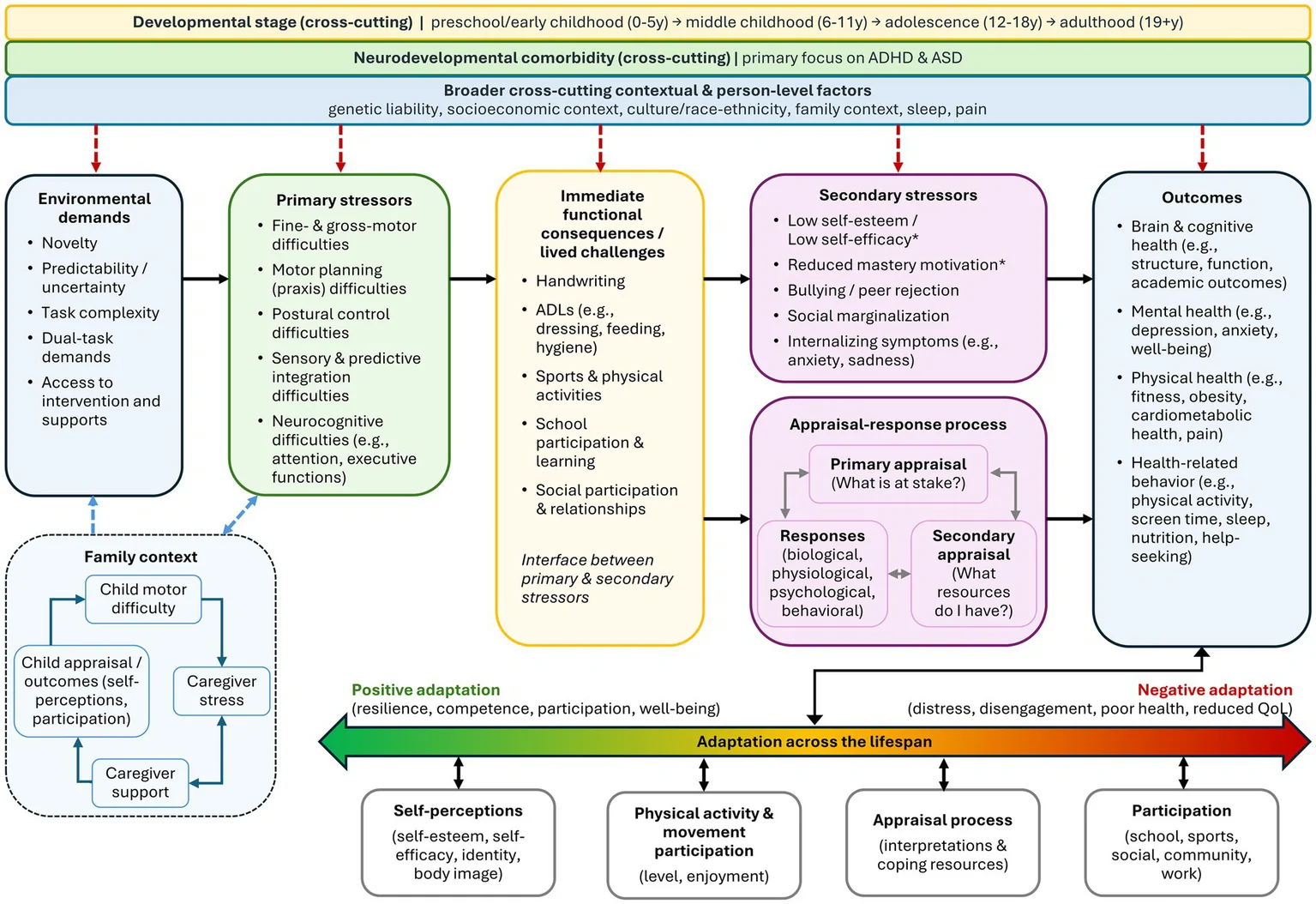
Integrated stress-adaptation framework for understanding mental health in developmental coordination disorder (DCD) and comorbid neurodevelopmental conditions (ISAF-DCD). Solid arrows indicate hypothesized directional pathways, and their evidential strength varies by pathway. The figure’s line and arrow styles distinguish *pathway type* (directional vs. speculative) but do not themselves encode the finer distinction between DCD-specific, indirect/extrapolated, and purely theoretical evidence; that distinction is deliberately reserved for Section 2.11, which should be consulted alongside the figure before any single pathway’s evidential weight is interpreted. Dashed red arrows indicate speculative links or emerging corticolimbic links, which are treated as a subordinate, exploratory extension of the framework rather than as part of its evidential core (Section 2.5). Developmental stage, neurodevelopmental comorbidity (ADHD and ASD), and a broader band of cross-cutting contextual and person-level factors (genetic liability, socioeconomic context, culture/race-ethnicity, family context, sleep, pain) are represented as three conceptually distinct cross-cutting bands (Section 2.1). Immediate functional consequences (e.g., handwriting, ADLs, sports, participation) form an interface between primary motor/neurocognitive stressors and secondary psychosocial stressors. Secondary stressors interact with cognitive appraisal and multilevel stress responses, which in turn influence outcomes in brain and cognitive health, mental health, physical health, and health-related behavior. Family-level reciprocity links child motor difficulties to caregiver stress and support (dashed blue arrows: Family context influences [bidirectional]). Adaptation across the lifespan reflects bidirectional pathways between outcomes, self-perceptions, physical activity, appraisal, and participation. Constructs marked with an asterisk occupy more than one functional role depending on measurement timing.

### 2.2 Primary stressors

Primary stressors in ISAF-DCD are the motor and neurocognitive difficulties associated with DCD, including fine- and gross-motor impairment, motor-planning difficulties, postural-control problems, and difficulties integrating sensory and cognitive information. These difficulties produce immediate functional consequences, such as poor handwriting or clumsiness. Conceptually, these consequences are best understood as the lived interface between underlying impairment and subsequent psychosocial experiences, rather than as belonging to a single category. They can give rise to secondary stressors, including low self-esteem, reduced self-efficacy, reduced mastery motivation, and bullying (Subara-Zukic et al., 2022).

#### 2.2.1 Motor difficulties

Characterizing motor impairment as a primary stressor is consistent with the ESH, which proposes that motor difficulties initiate a cascade of psychosocial consequences (Cairney et al., 2013; Li et al., 2018). Poor coordination can restrict participation in physical activity, academic tasks, and social interaction. Children with DCD may feel less competent than their peers and avoid activities that expose their difficulties, which may contribute to negative self-perceptions (Purcell et al., 2023; Southon, 2022; Weber et al., 2023). These findings support a plausible pathway from motor difficulty to psychosocial stress, but they do not, by themselves, establish a direct causal effect on mental health.

#### 2.2.2 Poor postural control

Children with DCD may show impaired postural stability, including greater postural sway in complex situations, altered anticipatory adjustments, and less efficient central and peripheral control (Godoi-Jacomassi et al., 2025). Within ISAF-DCD, these features are considered specific manifestations of motor impairment rather than independent psychological stressors.

#### 2.2.3 Motor planning and neurocognitive integration

Including motor planning and neurocognitive integration acknowledges that DCD involves more than motor-execution difficulties (Green and Dewey, 2026). Neuroimaging research has reported differences in the structure or activation of regions involved in motor control, including the cerebellum, basal ganglia, and parietal cortex (Subara-Zukic et al., 2022). Executive-function difficulties involving planning, organization, and cognitive flexibility are also reported in DCD and may compound the functional impact of motor impairment by increasing demands in academic and everyday contexts (Fogel et al., 2023; Lachambre et al., 2021; Zaguri-Vittenberg et al., 2026).

#### 2.2.4 Sensory and predictive processing

Emerging work suggests that differences in sensory processing and predictive motor control may contribute to difficulties with motor learning and adaptation to novel situations (Nobusako et al., 2018; Tal-Saban et al., 2025; Weber et al., 2025). At present, however, evidence directly linking these processes to mental-health outcomes in DCD is limited, and ISAF-DCD treats them as candidate mechanisms rather than established pathways.

#### 2.2.5 Immediate functional consequences

DCD can affect self-care tasks, such as tying shoelaces, fastening buttons, using utensils, and brushing teeth, as well as gross-motor activities, including balance, running, cycling, and learning complex movement sequences. Academic and occupational tasks may be affected through difficulties with handwriting or using tools, keyboards, and other equipment (Cairney, 2015; Green and Dewey, 2026; Sauer et al., 2026). These functional consequences mark points where underlying motor or neurocognitive difficulties become lived challenges. Accordingly, they occupy an explicit interface between primary and secondary stressors in the framework. Given the heterogeneity of DCD, individuals will not experience the same primary stressors with the same intensity. Proposed motor profiles involving manual dexterity, balance, planning, and visuoperceptual processing should therefore be treated as dimensional and provisional (Lust et al., 2022; Verbecque et al., 2026).

### 2.3 Secondary stressors

ISAF-DCD defines secondary stressors as psychosocial experiences that may arise from primary motor difficulties. Examples include low self-esteem, reduced mastery(motivation), diminished self-efficacy, bullying, peer rejection, and social marginalization. These experiences typically develop over time and interact with appraisal and coping processes rather than forming a simple, linear sequence.

#### 2.3.1 Self-esteem and self-efficacy

Children and adults with DCD have been reported to have lower self-esteem and self-efficacy (Cavalcante-Neto et al., 2026; Harris et al., 2025; Medeiros et al., 2023). Repeated motor failure and negative social comparisons may contribute to these outcomes, while global self-worth and movement-specific self-efficacy may mediate associations between motor difficulty and internalizing symptoms (Harris et al., 2025; Li et al., 2018). These constructs, therefore, have dual roles: they can be consequences of motor-related stress and mechanisms through which that stress affects later outcomes. Negative self-perceptions may also promote activity avoidance, limiting further skill development and social participation (Cavalcante and Draghi, 2021). This pattern suggests a reciprocal relationship rather than a one-way pathway.

#### 2.3.2 Mastery (motivation)

Mastery refers broadly to the perceived ability to influence and manage one’s environment and life outcomes (Bandura, 1997). Mastery motivation denotes the tendency to persist independently and effortfully when solving a problem, learning a skill, or completing a challenging task (Morgan et al., 1990). Although mastery motivation is conceptually relevant to DCD, it has received less direct study than self-esteem and self-efficacy. Successful persistence may strengthen mastery motivation and encourage engagement with increasingly challenging activities, whereas repeated experiences of uncontrollable motor failure may contribute to helplessness or reduced persistence. It remains unclear whether mastery motivation functions primarily as a secondary stressor, a mediator, a moderator, or a developmental process that changes in response to intervention. Evidence from young children at risk for developmental delay provides a useful conceptual foundation but should not be treated as direct evidence specific to DCD (Blasco et al., 2023; Dunford et al., 2005).

#### 2.3.3 Bullying and social acceptance

Bullying, peer rejection, and social marginalization are important potential social consequences of motor difficulty (Abregú-Crespo et al., 2024). Children with DCD may report fewer friendships, more bullying, and lower confidence in peer activities. From a stress perspective, peer rejection and bullying are environmental stressors, whereas shame, perceived exclusion, and reduced belonging reflect internalized meanings attached to those experiences. Qualitative studies suggest that stigmatizing labels and negative self-concepts may contribute to anxiety and depression during adolescence (Caçola, 2016; Cairney, 2018; Mancini et al., 2024).

#### 2.3.4 Internalizing problems

Internalizing problems include inwardly directed emotional distress, such as anxiety, depressive symptoms, somatic complaints, and social withdrawal (Carragher et al., 2015). Within ISAF-DCD, these problems may emerge in response to motor challenges, peer difficulties, reduced participation, and repeated failure. Once established, they may also influence later appraisal and coping. For example, anxiety may increase threat perception in motor situations, whereas depression may reduce motivation and energy for motor practice and social engagement (Draghi et al., 2020, 2021). Internalizing problems are therefore positioned in multiple ways within the framework, as possible responses, secondary stressors, mediators, and long-term outcomes, and their role should be interpreted with reference to measurement occasion and developmental stage.

### 2.4 Personal, biological, and social mediators and moderators

ISAF-DCD identifies several factors that mediate or moderate relationships between stressors and outcomes. A moderator is conceptualized as a pre-existing variable that changes the strength or direction of a relationship without itself being altered by the stress process (e.g., a trait-like disposition). A mediator sits on the causal pathway, is affected by the stressor, and in turn affects the outcome. These definitions are meaningful only when temporal ordering is specified; a variable cannot be identified as a mediator solely because it is associated with both an exposure and an outcome. Within ISAF-DCD, personality, trait resilience, social support, and access to intervention are treated primarily as moderators. Physical activity is treated as both a candidate mediator and a moderator because lower activity may result from motor difficulties and influence mental health outcomes (Li et al., 2018; Purcell et al., 2023).

#### 2.4.1 Moderators

##### 2.4.1.1 Resilience and personality

Personality and resilience may shape how individuals respond to motor difficulties (Black et al., 2024). In adults with DCD, higher resilience has been associated with greater self-efficacy and lower anxiety (Harris et al., 2024). Resilience can thus be conceptualized both as a relatively stable characteristic that moderates responses and as a dynamic process that develops through coping and adaptation (Idan et al., 2022). This distinction should be operationalized carefully: temperament or personality may precede a stress response, whereas resilience-related behaviors may strengthen with successful adaptation. Neurobiological markers of resilience, such as corticolimbic structure–function coupling, are potentially relevant but are largely derived from research outside DCD populations (Sisk et al., 2026).

The personality dimensions most relevant to adaptation in DCD remain uncertain. A study comparing neurodivergent groups found no significant association between DCD and extraversion, neuroticism, conscientiousness, or openness, but reported lower agreeableness among participants with formally diagnosed autism and DCD (Curșeu and van Rijswijk, 2026). Interpreting this finding requires caution because group composition, diagnostic status, and measurement characteristics may influence the result. Future research should identify which characteristics, such as optimism, conscientiousness, or neuroticism, are specifically associated with risk or protection in DCD.

##### 2.4.1.2 Social support

Family, peer, and teacher support may buffer the psychological effects of motor difficulties. During childhood, parental and teacher support may moderate relationships between environmental demands and stress responses (Mancini et al., 2024). Children with DCD who receive stronger support may experience fewer adverse psychological consequences, although DCD-specific evidence on the mechanisms remains limited (Caçola, 2016). As individuals mature, perceived support may also influence appraisal by changing whether a challenge is interpreted as threatening or manageable. Social support may thus be an important protective resource whose effects depend on its quality, timing, and accessibility (De Roubaix et al., 2025). Support also operates within families. Elevated parental stress suggests that relationships between child and caregiver stress are reciprocal rather than unidirectional (Cleaton et al., 2019; Jijon and Leonard, 2020). ISAF-DCD accordingly includes a family-level feedback loop in which child motor difficulty influences caregiver stress and caregiver stress and support influence child appraisal, participation, and outcomes.

##### 2.4.1.3 Access to interventions

Access to early identification, assessment, and intervention may alter developmental pathways. DCD remains under-recognized and underdiagnosed, and many individuals lack appropriate services (Klein et al., 2024; Meachon et al., 2024; Steenbergen et al., 2024). Access to intervention may moderate associations by altering the strength of existing pathways and serve as an intervention exposure when it directly changes motor, psychosocial, or mental-health outcomes.

#### 2.4.2 Mediators

##### 2.4.2.1 Physical activity

Physical activity may mediate associations between DCD and internalizing problems and may also moderate those associations by reducing adverse effects (Li et al., 2018). Children with DCD may be at increased risk of inactivity, creating a reciprocal cycle in which motor difficulties contribute to activity avoidance, which, in turn, limits skill development and may contribute to obesity and reduced fitness (Caçola, 2016; James et al., 2022; Li et al., 2025; Purcell et al., 2023). Poor health may reduce the capacity or motivation to participate, whereas appropriately adapted physical activity may improve physical and psychological outcomes. Longitudinal studies are needed to distinguish mediation from correlation and to identify the direction and timing of these effects (Stults-Kolehmainen and Sinha, 2014).

##### 2.4.2.2 Self-efficacy and self-esteem

Self-efficacy and self-esteem may mediate associations between motor performance and internalizing symptoms, anxiety, and physical activity. Lower motor performance levels may undermine self-perceptions, contributing to emotional distress and social withdrawal (Medeiros et al., 2023). Because self-efficacy and self-esteem are also treated as secondary stressors, ISAF-DCD models them as time-dependent constructs whose functional roles change across the course of adaptation.

##### 2.4.2.3 Executive function

Executive-function difficulties may help explain how motor-related stress relates to broader health outcomes. In children and adults with DCD, difficulties with planning, organization, shifting, and self-monitoring have been associated with internalizing symptoms, depression, and poorer well-being (Broletti et al., 2024). Executive-function difficulties may increase the effort required for routines, coping, and goal-directed behavior, thereby amplifying the impact of daily demands (Omer and Leonard, 2021). However, the direction of this pathway remains uncertain: executive-function differences may be a feature of DCD, a mediator of later outcomes, a consequence of chronic stress, or some combination of these processes. Longitudinal measurement is required to distinguish these possibilities and to specify, within ISAF-DCD, which measurement occasions and causal questions justify treating executive function as a stressor, mediator, or outcome.

##### 2.4.2.4 Internalizing problems

Internalizing symptoms may also mediate later outcomes. In adults with DCD, depression has been reported to mediate the relationship between coordination severity and quality of life, while emotional distress and self-efficacy jointly mediate associations between motor coordination and quality of life (Engel-Yeger and Engel, 2023; Engel-Yeger, 2025). In children, everyday executive-function difficulties may be associated with poorer well-being via internalizing symptoms (Omer and Leonard, 2021). These findings are compatible with chained mediation, but ISAF-DCD should treat specific sequences, for example, executive-function differences preceding internalizing symptoms, which in turn precede later quality of life, as developmental hypotheses that require longitudinal testing rather than as confirmed temporal pathways.

#### 2.4.3 Cross-cutting factors

##### 2.4.3.1 Developmental stage

Developmental stage is better represented as a cross-cutting factor rather than a discrete node because it may modify the salience and operation of each component. It may influence which primary stressors are most prominent, how visible secondary stressors become, how appraisal processes develop, which coping resources are available, and how outcomes are consolidated. For example, motor novelty may be especially salient in early childhood, whereas academic, athletic, occupational, and social demands may become more prominent later. Peer comparison may intensify during middle childhood and adolescence, while coping becomes increasingly self-directed (Sisk and Gee, 2022).

##### 2.4.3.2 Neurodevelopmental comorbidity

ISAF-DCD distinguishes neurodevelopmental comorbidity (ADHD and ASD) from a separate band of broader cross-cutting contextual and person-level factors, comprising genetic liability, socioeconomic context, culture and race/ethnicity, family context, sleep, and pain. These variables differ conceptually from comorbidity: they are not additional diagnoses but background conditions that may shape exposure, appraisal, or resilience across several components of the framework simultaneously, and future applications of ISAF-DCD should specify, for each variable, whether it is expected to operate as a moderator, a confounder of comorbidity effects, or an independent cross-cutting influence. DCD commonly co-occurs with ADHD and may co-occur with ASD, although estimates vary according to sampling, diagnostic criteria, and the use of screening versus clinical diagnoses (De Melo et al., 2026). In one SPARK-cohort analysis, 85% of children with ASD scored in the at-risk range on a DCD screening measure, whereas only 14% had a formal DCD diagnosis (Ketcheson et al., 2021). This contrast illustrates the importance of distinguishing screening status from diagnosis (De Melo et al., 2026). Genetic research indicates shared liability between DCD and ADHD, and children with both conditions may show greater depressive symptomatology than children with either condition alone (Martin et al., 2006; Piek et al., 2007). These findings support treating comorbidity as a potentially important moderator or cross-cutting condition. They do not, however, establish that comorbidity produces a distinct phenotype in every case or that its effects are multiplicative. Comorbidity may add attentional, executive, sensory, or social-communication demands; alter appraisal and coping; reduce access to activities or support; and increase outcome severity.

### 2.5 Appraisal, response, and interactive dynamic processes

The central component of ISAF-DCD depicts an interaction among cognitive appraisal, possible neurobiological correlates, coping efforts, stress responses, and subsequent appraisal. The response domains include biological, physiological, psychological, and behavioral processes. I use “biological” to refer to molecular, cellular, and neuroanatomical substrates (e.g., HPA-axis activity, corticolimbic structure), and “physiological” to refer to their more directly observable, whole-organism correlates (e.g., heart-rate variability, cortisol output, motor tremor); the two are related but not interchangeable, and studies that measure one should not be assumed automatically to index the other. Bidirectional arrows represent an iterative process rather than a linear sequence, emphasizing that appraisals and responses mutually influence one another over time.

#### 2.5.1 Cognitive appraisal

The inclusion of cognitive appraisal follows the transactional model of stress, which distinguishes primary appraisal of threat or harm from secondary appraisal of coping resources (Lazarus and Folkman, 1984). In DCD, a motor challenge may be appraised as threatening, harmful, challenging, or controllable. Research suggests that some individuals with DCD experience movement-related anxiety and reduced self-efficacy (Harris et al., 2024, 2025). Perceived lack of control is therefore a plausible appraisal variable, particularly when motor performance is unpredictable. At the same time, not all individuals with DCD appraise motor situations as threatening; appraisal likely varies with developmental stage, prior experience, task demands, social context, self-efficacy, and available support.

#### 2.5.2 Corticolimbic system (a subordinate, exploratory hypothesis)

I wish to be explicit that the psychosocial components of ISAF-DCD (i.e., appraisal, secondary stressors, coping, and social process) constitute the framework’s evidential core. In contrast, the proposed corticolimbic substrate is a subordinate and considerably more speculative extension that is not required for the rest of the framework to hold. I include it because a biologically plausible mechanism is theoretically useful, not because direct evidence currently supports it in DCD. The proposed link between appraisal and corticolimbic systems draws on the broader literature implicating these systems in threat processing, emotion regulation, and stress adaptation (Smith and Pollak, 2020). Early-life stress has been associated with changes in prefrontal, hypothalamic, amygdala, and dopaminergic systems, partly through alterations in hypothalamic–pituitary–adrenal (HPA) axis functioning (Smith and Pollak, 2020), and chronic psychosocial stress has also been associated with structural and functional changes in corticolimbic networks (Demartini et al., 2020; Sisk et al., 2026). However, the specific pathway from these general findings to motor planning or motor execution in DCD remains untested. In ISAF-DCD, the suggestion that altered top-down control or a stress-related freezing response disrupts movement timing is therefore explicitly framed as a hypothesis rather than an established mechanism. Similarly, evidence of HPA-axis dysregulation in ADHD (Fulun et al., 2025) cannot be treated as direct evidence of the same process in DCD, and neurobiological differences reported in DCD (Scrocco et al., 2026) do not, by themselves, demonstrate altered stress appraisal or explain mental-health outcomes. Readers unconvinced by the corticolimbic component can set it aside without weakening the psychosocial pathways that carry most of the framework’s current empirical weight; this dependency structure is reflected in the evidence map (Section 2.11).

#### 2.5.3 Response domains

Four response domains recognize that stress responses may occur across several systems. Biological responses may include changes in cortisol or autonomic regulation, physiological responses may include muscle tension, sweating, or gastrointestinal discomfort, psychological responses may include embarrassment, frustration, or anxious anticipation, and behavioral responses may include freezing, task avoidance, or compensatory over-effort, which can increase error rates (Harris et al., 2024; Smith and Pollak, 2020). These domains are interdependent in practice, but distinguishing them is useful because self-report, physiological measures, and observed behavior may capture different parts of the process. Future studies should specify which response domain they measure and how measurement timing relates to appraisal, stressor exposure, and subsequent outcomes.

#### 2.5.4 Bidirectional dynamics

Bidirectional arrows between appraisal and response are consistent with an iterative stress process in which initial appraisals influence responses, and responses, in turn, shape later appraisals. For example, avoidance may reduce anxiety in the short term while reinforcing threat expectations in future situations (Jacobson and Newman, 2014). The same logic suggests that links between the interactive appraisal-response process and outcomes should be reciprocal. Depression may increase perceived lack of control and reduce physical activity, while repeated motor-related stress may contribute to depression (Douka et al., 2025). Most DCD studies to date have focused on outcomes rather than on the cognitive and neurobiological processes proposed to generate them. Consequently, the appraisal-response component of ISAF-DCD rests on limited direct evidence, and the framework explicitly treats these links as hypotheses to be tested. Research should examine whether developmental stage, motor profile, ADHD, and ASD are associated with distinct appraisal and response patterns.

### 2.6 Short-term and long-term outcomes

ISAF-DCD distinguishes short-term functioning from longer-term outcomes. Short-term outcomes include negative functioning, such as poor motor performance, anxiety, and withdrawal, and positive functioning, such as engagement, successful performance, and well-being. Longer-term outcomes include brain and cognitive health, mental health, physical health, and health-related behavior. Conceptually, short-term functioning should be treated as an intermediate state that may serve as an outcome at one time point, a response at another, or a mediator across adjacent time points, depending on the causal question. This distinction extends the ESH by recognizing that motor difficulties may be associated with several downstream domains, not only internalizing problems.

#### 2.6.1 Brain and cognitive health

Including brain and cognitive outcomes, including executive function, reflects evidence that chronic stress can affect brain development and cognition (Antolini and Colizzi, 2023). Executive-function difficulties have been reported as mediators of associations between DCD-related symptoms and depression in adults (Broletti et al., 2024). Within ISAF-DCD, these mediation claims are provisional, pending longitudinal evidence demonstrating the temporal ordering of motor symptoms, executive differences, and depression.

#### 2.6.2 Mental health

Anxiety and depression are consistently reported as elevated in DCD populations. A systematic review and meta-analysis found moderately higher internalizing symptoms than in typically developing peers (Omer et al., 2019). Individual studies have reported higher rates of clinical anxiety and depression among children and adolescents with DCD, although estimates vary substantially across samples and measures (Harrowell et al., 2017). Loneliness has received less direct study than anxiety and depression, but it remains a plausible outcome of bullying, peer rejection, and reduced participation. Adults with DCD report mood difficulties and reduced quality of life relative to comparison groups (Cavalcante-Neto et al., 2026; Hill and Brown, 2013).

#### 2.6.3 Physical health

DCD may be associated with reduced fitness, lower endurance, obesity, and persistent difficulties with ADL and self-care (Caçola, 2016). A systematic review reported large group differences in aerobic, anaerobic, and muscular strength (Cavalcante Neto et al., 2024). A meta-analysis reported higher odds of overweight or obesity among individuals with DCD, with stronger associations in older samples (Gambra et al., 2024). These associations may be partly explained by physical inactivity and sedentary behavior, but establishing causal pathways requires longitudinal evidence (Purcell et al., 2023).

#### 2.6.4 Health-related behavior

Physical inactivity and sedentary behavior are considered outcomes because motor difficulties may reduce participation in physical activity. They may also contribute to later physical and mental-health problems, making them part of a reciprocal process rather than a final endpoint (Caçola, 2016; Li et al., 2018). ISAF-DCD therefore treats health-related behavior as a domain in which variables such as physical activity can serve as outcomes at one point in time and as mediators or feedback mechanisms at another.

### 2.7 Adaptation across the lifespan

ISAF-DCD includes a bidirectional adaptation pathway, indicating that outcomes may be positive or negative and that adaptation continues across the lifespan. This feature is important because DCD does not produce a uniform developmental course. Some individuals experience persistent or accumulating difficulties, whereas others develop effective compensatory strategies and maintain positive well-being (Meachon and Alpers, 2023). Positive adaptation may be associated with cognitive resources, academic functioning, social skills, supportive relationships, and the absence of bullying (Steenbergen et al., 2024). These factors are treated as candidate protective processes rather than universal predictors, and the framework remains agnostic about which combinations are necessary or sufficient for resilience. Resilience research also points to cognitive reframing, compensatory strategies, and identity development as possible mechanisms of successful adaptation. In ISAF-DCD, positive adaptation can be conceptualized as an outcome, a process, or a higher-order developmental trajectory. For clarity, future ISAF-DCD applications should specify which meaning each analysis intends. Feedback from outcomes to earlier components is theoretically important. For example, depression may increase perceived lack of control, reduce physical activity, and intensify social isolation. These feedback pathways remain underexplored in DCD and should be tested using repeated longitudinal assessments.

### 2.8 Developmental-stage-specific configurations of ISAF-DCD

Because the causal components of ISAF-DCD are largely developmentally general, the developmental-stage axis requires age-specific hypotheses to become empirically useful. The relative salience of each component may change across development, and Table 1 translates these propositions into testable developmental hypotheses.

**Table 1:** Hypothesized developmental salience of ISAF-DCD components (qualitative).

Component	Preschool/early childhood	Middle childhood	Adolescence	Adulthood
Primary motor stressors	High	Moderate–High	Moderate–High	Low–Moderate
Secondary stressors	Low	High	High	Moderate–High
Appraisal/response processes	Low–Moderate	Moderate–High	High	High
Mediators (e.g., self-efficacy, EF)	Moderate–High	Moderate–High	High	High
Moderators (e.g., support, comorbidity)	Low–Moderate	Moderate–High	High	High
Outcomes (mental, cognitive, physical, behavioral)	Low	Moderate–High	High	High

Categories represent hypothesized relative emphasis, not measured effect sizes or standardized scores, and are intended to guide age-stratified empirical testing rather than to summarize existing quantitative findings.

In preschool and early childhood, motor difficulties may be less socially visible when expectations and peer comparison are limited, yet young children with motor delays have shown emotional and behavioral difficulties, suggesting that stress-related processes may begin early (Dreiskämper et al., 2022; Rodriguez et al., 2019). Early pathways may involve frustration, caregiver stress, and activity avoidance (Jijon and Leonard, 2020). In middle childhood, motor difficulties may become more apparent during physical education, sports, playground activities, and peer comparisons. Bullying and social exclusion may become more prominent, potentially contributing to secondary stressors and internalizing symptoms (Abregú-Crespo et al., 2024).

During adolescence, motor difficulties intersect with identity development, peer relationships, intimate relationships, and increasing demands for independence. Adolescents with DCD may face increased mental-health risk, and some evidence suggests particular vulnerability among females (Erskine et al., 2024; Fortnum et al., 2026). Greater executive and social demands may also increase the consequences of motor and cognitive difficulties. In adulthood, relevant demands include employment, education, independent living, and intimate relationships. Adults with DCD continue to report depression, anxiety, and reduced quality of life, while occupational and educational difficulties may undermine self-esteem (Engel-Yeger and Engel, 2023; Verlinden et al., 2023). Some adults develop compensatory strategies and report successful adaptation (Rathbone and Wilmut, 2025). Table 1 translates the developmental narrative above into qualitative, stage-specific hypotheses about the relative salience of six ISAF-DCD components.

This pattern generates several directly testable, stage-specific predictions. First, I predict that the *rate of increase* in secondary-stressor salience (e.g., peer rejection, low self-esteem) from preschool to middle childhood will exceed the rate of increase in primary motor-stressor salience over the same period, reflecting a shift from direct motor difficulty toward its social and evaluative consequences. Second, I predict that appraisal-related constructs (self-efficacy, perceived control) will show larger longitudinal associations with internalizing symptoms in adolescence and adulthood than in early childhood, when appraisal capacity is less developed. Third, I predict that moderators such as social support and access to intervention will account for a larger proportion of outcome variance from middle childhood onward, once secondary stressors become more salient, than in preschool. These predictions could be tested using age-stratified or longitudinal cohorts that assess motor difficulty, self-efficacy, peer relationships, and mental-health outcomes at repeated occasions (see Section 5).

### 2.9 Clarifying temporal and functional roles of dual-role constructs

Several constructs (e.g., self-esteem/self-efficacy, internalizing symptoms, executive function, physical activity, resilience, and mastery motivation) can occupy multiple positions in ISAF-DCD depending on developmental timing and analytic context. A dynamic framework can legitimately allow such time-varying roles, but without specifying the conditions under which each role applies, the framework risks becoming unfalsifiable: almost any empirical pattern could be absorbed *post hoc* by relabeling a construct’s function. Table 2 makes these conditions explicit for the six constructs, following the general schema construct → measurement occasion/temporal condition → proposed functional role → predicted downstream relationship.

**Table 2:** Temporal and functional roles of key dual-role constructs in ISAF-DCD.

Construct	Measurement occasion/temporal condition	Proposed functional role	Predicted downstream relationship
Self-esteem/self-efficacy	Measured at baseline, before repeated motor-related failure	Moderator	Buffers or amplifies the effect of motor difficulty on later internalizing symptoms
	Measured after repeated motor-related failure or negative feedback	Mediator (secondary stressor)	Transmits the effect of motor difficulty onto later internalizing symptoms and reduced participation
Internalizing symptoms	Measured concurrently with peer rejection or bullying	Response / secondary stressor	Co-occurs with, and may reciprocally intensify, social secondary stressors
	Measured at a later developmental stage than motor difficulty	Outcome	Represents a downstream mental-health consequence of the cumulative pathway
Executive function	Measured in early-to-middle childhood, alongside motor assessment	DCD-related characteristic / primary-stressor correlate	Contributes jointly with motor difficulty to immediate functional consequences
	Measured in adolescence/adulthood, after motor and social stressors have accumulated	Mediator	Transmits cumulative stress exposure onto reduced academic or occupational outcomes
Physical activity	Measured as a stable trait-like pattern across childhood	Mediator	Transmits the effect of motor difficulty on physical-health and mental-health outcomes via reduced participation
	Measured as a moderating pre-existing engagement level	Moderator	Buffers the effect of motor difficulty on negative adaptation (higher baseline activity attenuates negative outcomes)
Resilience	Measured as a pre-existing person-level resource	Moderator	Buffers the pathway from secondary stressors to negative adaptation
	Measured as a lifespan outcome, following successful adaptation	Outcome	Represents a positive-adaptation endpoint on the lifespan adaptation continuum
Mastery motivation	Measured before a specific motor task	Moderator of appraisal	Shapes whether a motor challenge is appraised as a threat or a challenge
	Measured as declining following repeated failure	Secondary stressor / mediator	Transmits cumulative motor difficulty onto reduced engagement and negative adaptation

This table does not exhaust every plausible role a construct might play; rather, it operationalizes the framework’s central dynamic claim well enough that specific studies can pre-register which role they are testing, which is a precondition for falsifiability. I recommend that future empirical applications of ISAF-DCD state in advance which row of Table 2 (or an explicitly justified extension of it) their design tests.

### 2.10 Core falsifiable propositions of ISAF-DCD

A conceptually integrative framework is not automatically distinguishable, in its empirical implications, from an expanded Environmental Stress Hypothesis (ESH) or from its constituent stress theories considered separately. To address this concern directly, I specify five core propositions that (a) follow from the structure of ISAF-DCD, (b) are, in principle, empirically distinguishable from the predictions of an expanded ESH or of the constituent theories alone, and (c) are stated at a level of specificity that permits disconfirmation.

#### P1 (developmentally specific mediation)

The indirect effect of motor difficulty on internalizing symptoms via self-esteem/self-efficacy will be significantly stronger in middle childhood and adolescence than in preschool, because self-evaluative processes and peer comparison are not yet fully developed in early childhood. An expanded ESH that does not explicitly model appraisal-mediated pathways predicts a developmentally constant or monotonically increasing mediation effect rather than this specific non-linear, stage-dependent pattern.

#### P2 (comorbidity × pathway interaction)

Comorbid ADHD will amplify the primary-stressor-to-secondary-stressor pathway (via increased functional and attentional demands). In contrast, comorbid ASD will amplify the secondary-stressor-to-appraisal pathway (via differences in social appraisal and peer interaction), producing a dissociable pattern across comorbidity subgroups rather than a uniform severity effect predicted by models that treat comorbidity as a simple additive risk factor.

#### P3 (reciprocal family-context feedback)

Caregiver stress at one timepoint will predict child appraisal and motor-related avoidance at a later timepoint, and child motor difficulty and appraisal will reciprocally predict later caregiver stress, producing a bidirectional, cross-lagged association that a unidirectional stress-exposure model (including standard ESH) would not predict.

#### P4 (appraisal as a discriminating moderator)

Among individuals with comparable levels of objectively measured motor impairment, those with higher perceived control/self-efficacy will show meaningfully better mental-health outcomes than those with lower perceived control, and this appraisal-based moderation will account for outcome variance beyond that explained by motor severity alone - a prediction that follows from the transactional-appraisal component of ISAF-DCD but not from severity-based models of DCD impact.

#### P5 (non-motor secondary-stressor pathway)

Secondary stressors (peer rejection, low self-esteem) will predict later internalizing symptoms even after statistically controlling for concurrent motor severity, indicating that the psychosocial pathway carries incremental predictive value beyond motor impairment itself, which is the central claim distinguishing ISAF-DCD from purely motor-deficit accounts of mental-health risk in DCD.

These propositions do not capture the full implications of ISAF-DCD. However, they constitute a minimal set of empirically tractable, stage-appropriate hypotheses whose disconfirmation would require meaningful revision of the framework, and whose confirmation would provide evidence for ISAF-DCD’s added value relative to simpler alternatives.

### 2.11 Evidence map for major pathways

Table 3 summarizes, for each major pathway discussed in Sections 2.1–2.7, whether the supporting evidence is DCD-specific or indirect/extrapolated, what type of evidence is available, its current evidential status, and the key empirical prediction that remains to be tested. This map is not a substitute for a systematic review, but it is intended to make the evidential heterogeneity of ISAF-DCD explicit and navigable.

**Table 3:** Evidence map for major ISAF-DCD pathways.

Pathway/construct	DCD-specific or indirect	Evidence type	Evidential status (representative sources)	Key untested prediction
Motor difficulty → primary stressors	DCD-specific	Cross-sectional, some longitudinal	Relatively well supported (Cairney et al., 2013; Subara-Zukic et al., 2022)	Dose–response relationship between severity and stressor accumulation across stages
Primary stressors → secondary stressors (peer rejection, low self-esteem)	DCD-specific	Cross-sectional, longitudinal	Relatively well supported (Cairney et al., 2010; Li et al., 2018; Rodriguez et al., 2019)	Temporal ordering and reciprocal effects using cross-lagged designs
Secondary stressors → internalizing symptoms	DCD-specific, some indirect (broader stress literature)	Cross-sectional, some longitudinal	Moderately supported (Harris et al., 2025; Omer et al., 2019)	Incremental prediction beyond concurrent motor severity (P5)
Appraisal (self-efficacy/control) → outcomes	Indirect (extrapolated from broader appraisal literature; limited DCD-specific data)	Cross-sectional, theoretical	Theoretical/provisional (Harris et al., 2024, 2025; Lazarus and Folkman, 1984)	Appraisal as discriminating moderator among equally impaired individuals (P4)
Comorbidity (ADHD/ASD) × pathway differentiation	DCD-specific but limited	Cross-sectional	Theoretical/provisional (Martin et al., 2006; Mountford et al., 2021; Piek et al., 2007)	Dissociable amplification patterns across comorbidity subgroups (P2)
Family-context reciprocal loop	Indirect (extrapolated from general parenting-stress literature)	Cross-sectional, limited longitudinal	Theoretical/provisional (Cleaton et al., 2019; Jijon and Leonard, 2020)	Bidirectional cross-lagged association between caregiver stress and child appraisal (P3)
Corticolimbic/HPA-axis mechanisms	Indirect (extrapolated from general stress neuroscience and ADHD literature)	Theoretical, animal/general-population evidence	Speculative (Demartini et al., 2020; Fulun et al., 2025; Sisk et al., 2026; Smith and Pollak, 2020)	Direct psychophysiological or neuroimaging test of stress-related motor disruption in DCD
Physical activity as mediator/moderator	DCD-specific, mixed	Cross-sectional, some longitudinal	Moderately supported (Li et al., 2018; Purcell et al., 2023; Stults-Kolehmainen and Sinha, 2014)	Discrimination between mediating and moderating roles using the schema in Table 2
Developmental-stage salience pattern (Table 3)	Indirect (extrapolated from cross-sectional, cross-stage comparisons)	Theoretical, cross-sectional inference	Theoretical/provisional (Dreiskämper et al., 2022; Erskine et al., 2024; Piek et al., 2007; Rasmussen and Gillberg, 2000)	Age-stratified longitudinal test of the stage-specific predictions in Section 2.8

## 3 Integrative discussion of the overall model

### 3.1 Causal directionality and shared vulnerabilities

A central question for ISAF-DCD is whether motor difficulties causally precede mental, cognitive, and physical health problems or whether observed associations reflect shared neurodevelopmental vulnerabilities and reciprocal influences.

The ESH proposes that motor impairment initiates a sequence of psychosocial consequences that increase the risk of mental-health problems (Cairney et al., 2013). Sequential mediation studies have linked DCD-related motor difficulty with physical inactivity and lower global self-worth, which, in turn, are associated with internalizing symptoms (Cairney et al., 2010; Li et al., 2018). Longitudinal evidence that early motor skills predict later anxiety and depressive symptoms is also consistent with a developmental cascade (Rodriguez et al., 2019; Schneider and West, 2025). These findings support a plausible experiential pathway. However, ISAF-DCD emphasizes that cross-sectional mediation should not be interpreted as definitive evidence of temporal causation; confounding and shared liability remain viable alternative explanations.

Alternative interpretations challenge a purely causal account in which motor difficulties alone drive later mental-health problems. Twin and sibling research indicates that familial genetic and shared environmental factors may account for a substantial part of the association between coordination difficulties and internalizing symptoms, with nonshared environmental influences accounting for approximately one-third of the phenotypic association in one study (Waszczuk et al., 2016). Shared genetic liability between DCD and ADHD, together with associations between motor coordination, copy-number-variant risk, and psychopathology, further supports a common-vulnerability account (Martin et al., 2006; Mountford et al., 2021). A candidate neurodevelopmental mechanism for this shared vulnerability is corollary discharge (efference copy): the internal prediction, generated when a motor command is issued, that is normally used to distinguish self-generated from externally caused sensory events. Impaired corollary-discharge signaling has been proposed as a transdiagnostic mechanism linking motor abnormalities to psychosis-spectrum symptoms (Yao et al., 2024), and motor signs occurring years before symptom onset have been reframed as a potential transdiagnostic marker of broader psychopathological vulnerability rather than a schizophrenia-specific feature (Poletti and Raballo, 2022). Consistent with this, children at familial high risk of schizophrenia or bipolar disorder show impaired motor development relative to population controls, and this early motor impairment is associated with subsequent psychotic experiences (Burton et al., 2023). Although this literature concerns psychosis-spectrum and mood-disorder risk rather than DCD specifically, it raises the possibility that at least part of the association between motor difficulty and later psychopathology in ISAF-DCD reflects a shared, corollary-discharge-related neurodevelopmental substrate rather than (or in addition to) the experiential, stress-mediated pathway that is the framework’s main focus. I flag this as an alternative or complementary mechanism worth testing directly in DCD samples, for example by comparing sensory attenuation or corollary-discharge indices (such as saccadic suppression or self-generated touch attenuation) between children with and without DCD, and by testing whether such indices statistically account for any of the DCD-mental-health association independently of the appraisal- and stress-mediated pathways specified elsewhere in ISAF-DCD.

Relationships may also operate in both directions. Anxiety-related avoidance may reduce motor practice and social participation, while depression may reduce motivation for physical activity and worsen physical-health outcomes. ISAF-DCD acknowledges these feedback loops but notes that direct evidence for specific bidirectional pathways in DCD remains limited. A plausible integrative hypothesis is that several processes operate simultaneously: shared neurodevelopmental vulnerability contributes to initial co-occurrence; motor difficulties act as experiential stressors that may exacerbate mental-health problems; and mental-health problems feed back through behavioral and motivational pathways to affect motor participation and physical health. Longitudinal designs with repeated measures of motor performance, appraisal, stress, behavior, and mental health are needed to test this hypothesis.

### 3.2 Developmental trajectories

ISAF-DCD implies heterogeneity in developmental outcomes but does not yet specify empirically derived trajectories. Drawing on developmental psychopathology (Healy et al., 2022; Scheel Rasmussen et al., 2026), I hypothesize at least five trajectory patterns for DCD, while noting that direct empirical evidence identifying these specific patterns in DCD populations is currently lacking.

1. Resilient trajectory: relatively good mental health and successful adaptation maintained through protective factors such as strong social support, early intervention, compensatory strategies, and positive self-concept.
2. Improving trajectory: motor or functional difficulties decrease following intervention, secondary stressors diminish over time, and mental health and functioning improve.
3. Stable-difficulties trajectory: motor difficulties and psychosocial challenges persist but do not substantially worsen.
4. Deteriorating trajectory: secondary stressors accumulate, mental health worsens, and physical health problems develop over time.
5. Late-emerging-difficulties trajectory: motor difficulties are manageable during childhood but become more consequential when demands increase in adolescence or adulthood.

Candidate protective factors include social support, early intervention, good social skills, strong academic functioning, absence of bullying, resilience, and engagement in adapted physical activity. Candidate risk factors include severe motor impairment, ADHD or ASD, executive-function difficulties, early bullying, low self-esteem, inactivity, and limited access to services.

Longitudinal evidence specific to DCD remains limited. A 22-year follow-up of children with ADHD and DCD found that neurodevelopmental symptoms, including motor dysfunction, predicted adverse adult outcomes (Rasmussen and Gillberg, 2000). Twin research reported greater depressive symptomatology among children with co-occurring ADHD and DCD than among children with either condition alone (Piek et al., 2007). These findings support the importance of comorbidity and symptom severity but do not establish the five trajectories proposed above; ISAF-DCD thus treats trajectory patterns as hypotheses to be tested rather than as established typologies.

### 3.3 Is ISAF-DCD a heuristic, a hypothesis-generator, or a predictive theory?

The comprehensiveness that allows ISAF-DCD to integrate a fragmented literature also risks creating a framework that accommodates nearly every relevant construct and reciprocal pathway descriptively, while offering limited predictive constraint. I think it is more accurate, and more useful to readers, to state plainly that ISAF-DCD currently serves three distinguishable functions to different degrees, rather than claiming a single status for the whole framework. As an organizing framework that integrates the DCD, stress, and developmental-psychopathology literatures under a common structure, ISAF-DCD is, I believe, largely successful: Sections 2 and 4 demonstrate that most of its components have some grounding in existing DCD or adjacent literature. As a heuristic for generating developmental research questions, it is also useful, as illustrated by the stage-specific predictions in Section 2.8 and the trajectory hypotheses above. As a predictive theory whose propositions are stated with enough specificity to be disconfirmed independently of its constituent theories, ISAF-DCD is considerably less mature; the five core propositions in Section 2.10 and the construct-role table in Section 2.9 represent my attempt to move toward this third function, but they cover only a subset of the framework’s total content, and much of the remaining material (e.g., the full corticolimbic pathway, the complete family-context loop) remains at the heuristic rather than the predictive stage. I therefore recommend that readers and future users of ISAF-DCD treat Sections 2.9 and 2.10 as the framework’s current predictive core, and treat the remaining components as organizing and heuristic scaffolding that is available for, but has not yet achieved, comparable empirical specification.

## 4 Theoretical integration: relationship to established stress models

ISAF-DCD draws on several established stress theories. Presenting these theories from general models of vulnerability to DCD-specific accounts clarifies both the framework’s foundations and its proposed contribution.

Diathesis-stress models posit that psychopathology emerges from the interaction between pre-existing vulnerability (diathesis) and environmental stressors (Zuckerman, 1999; Luiz and Tsutsumi, 2026). In DCD, motor impairment may serve as a neurodevelopmental vulnerability that increases exposure to, or sensitivity to, environmental stress (Engel-Yeger and Engel, 2023; Rodriguez et al., 2019; Verlinden et al., 2023). ISAF-DCD extends this logic by distinguishing primary and secondary stressors, specifying mediating processes, and incorporating feedback loops. The neural diathesis-stress model of schizophrenia provides a useful comparison because it links stress, HPA-axis activity, neurodevelopmental vulnerability, and brain processes (Pruessner et al., 2017). However, evidence of altered HPA-axis functioning or stress physiology in DCD is limited, and evidence from ADHD should be treated as an indirect comparison rather than direct support for the DCD model (Fulun et al., 2025).

Differential-susceptibility models propose that some individuals are more responsive to both adverse and supportive environmental conditions (Broletti et al., 2024; Cairney, 2018). ISAF-DCD currently emphasizes risk and negative outcomes, but its inclusion of positive adaptation is compatible with differential-susceptibility hypotheses. Individuals who are particularly affected by adverse motor and social experiences may also benefit most from well-matched interventions and supportive environments. This possibility requires direct tests of interaction effects; it cannot be inferred from resilience-related outcomes alone.

The transactional model emphasizes appraisal and coping: stress depends not only on events but also on how individuals evaluate threat, controllability, and available coping resources (Lazarus and Folkman, 1984). ISAF-DCD incorporates primary appraisal of threat or harm and secondary appraisal of coping resources. The emphasis on perceived lack of control is especially relevant to DCD because unpredictable motor performance may increase perceptions of uncontrollability (Tal-Saban et al., 2012).

The Conservation of Resources model proposes that stress arises when valued resources are threatened, lost, or insufficiently replenished (Hobfoll, 1989). Resource loss can spiral because each loss reduces capacity to cope with subsequent demands. Within ISAF-DCD, motor difficulties may threaten resources for competence, autonomy, participation, and social acceptance. Secondary experiences, such as reduced self-esteem, mastery, and peer acceptance, may further deplete resources that could otherwise buffer stress. This perspective offers a testable account of why a secondary-stressor cascade may accelerate and suggests that interventions introduced before losses accumulate may be particularly effective. However, the proposed “resource-loss spiral” in DCD remains a hypothesis; researchers have not yet empirically demonstrated it in this population. Stress-generation theory provides a related behavioral mechanism: depression may generate additional stress through withdrawal, interpersonal conflict, or reduced engagement (Hammen, 1991; Santee et al., 2023). This process can be integrated with the resource-loss account, provided that the framework clearly distinguishes the theories rather than treating them as interchangeable.

The ESH is the most DCD-specific precursor of ISAF-DCD. It proposes that motor impairment acts as a primary stressor, leading to psychosocial consequences that increase the risk of internalizing problems (Cairney et al., 2013; Erskine et al., 2024; Li et al., 2018; Medeiros et al., 2022). ISAF-DCD retains the primary-secondary stressor distinction while extending it by incorporating appraisal, candidate neurobiological correlates, additional mediators and moderators, multiple long-term outcome domains, feedback loops, and adaptation.

Relative to the theories reviewed above, ISAF-DCD proposes five main contributions, which vary in how novel they are relative to the ESH and the constituent stress theories. Two are largely *adapted* from existing models: (1) integration of motor-specific stressors with general stress theory, which formalizes a synthesis implicit in prior DCD-stress writing; and (3) inclusion of cognitive, mental, physical, and behavioral outcomes, which extends the outcome range considered by the ESH without introducing a new mechanism. Three are, to my knowledge, more genuinely *novel* contributions of ISAF-DCD specifically: (2) explicit specification of the temporal and functional roles of dual-role DCD-relevant psychosocial constructs such as self-esteem, mastery, and internalizing symptoms (Table 2), which the ESH and related models do not attempt; (4) the reciprocal, feedback-based treatment of adaptation across the lifespan, including the family-context loop, rather than a unidirectional exposure-to-outcome sequence; and (5) explicit treatment of neurodevelopmental comorbidity as a cross-cutting, pathway-differentiating condition (P2) rather than as an additive severity factor. The five core propositions in Section 2.10 operationalize these contributions, particularly (2), (4), and (5), into empirically distinguishable predictions; where a contribution is more clearly an extension of existing theory (as in 1 and 3), I make no claim that ISAF-DCD’s predictions diverge from those of an expanded ESH. Taken together, these contributions make the framework a potentially useful organizing heuristic, but, as discussed in Section 3.3, they do not, by themselves, guarantee that all proposed pathways are empirically valid.

## 5 Recommendations for future research

Empirical research priorities include several domains. Longitudinal studies are needed to examine causal pathways and bidirectional relationships directly, rather than inferring directionality from cross-sectional associations. Age-stratified and repeated-measures designs should test whether the stage-specific pattern proposed in Table 1 holds empirically. Cohort studies following individuals from childhood into adulthood could identify predictors of resilient, improving, stable-difficulties, deteriorating, and late-emerging trajectories. Direct examination of cognitive appraisal, coping, autonomic activity, HPA-axis function, and neurobiological stress processes in DCD samples is required, particularly to test whether comorbidity-linked HPA-axis and executive-function vulnerabilities combine multiplicatively with DCD’s motor and cognitive profile, as Section 2.5 highlights as untested. Research on positive adaptation and compensatory mechanisms should draw on resilience work on cognitive reframing, strategy use, identity development, and person-environment fit. Intervention studies are needed to test whether modifying proposed mediators, such as physical activity, self-esteem, self-efficacy, social support, or threat appraisal, changes later outcomes and whether timing interventions earlier in the resource-loss cascade improves their effectiveness.

Methodologically, future studies should use repeated measures, clearly distinguish clinical diagnoses from screening classifications, and assess motor, psychological, social, behavioral, and physiological variables with instruments appropriate to each developmental stage. Mediation and moderation analyses should be embedded within longitudinal designs and interpreted cautiously. Where possible, studies should test alternative explanations involving shared genetic liability, socioeconomic context, comorbidity, and reciprocal effects.

ISAF-DCD also has clinical implications. It suggests several potential intervention targets: early identification and motor intervention; anti-bullying and social-participation programs; adapted physical-activity opportunities; support for self-efficacy and self-esteem; caregiver and teacher support; cognitive-behavioral approaches addressing threat appraisal and coping; and monitoring and treatment of emerging mental-health problems. These targets should not be interpreted as evidence that every individual with DCD requires multilayered treatment. Rather, the framework supports the individualized assessment of motor, environmental, psychosocial, and mental health needs. Early, accessible services may be particularly important given persistent under-recognition and underdiagnosis.

## 6 Conclusion

ISAF-DCD is a promising attempt to integrate empirical findings and established stress theories into a developmental model of how motor difficulties may relate to mental, cognitive, physical, and behavioral outcomes. Its principal strengths are its multilevel conceptualization of stressors, incorporation of appraisal and coping, attention to mediators and moderators, recognition of multiple outcome domains, and emphasis on reciprocal adaptation across the lifespan.

At the same time, the framework remains provisional. The present evaluation is narrative rather than systematic, and evidence selection may therefore be subject to bias. Some interpretations depend on the representation of the framework in Figure 1 and on the cited literature rather than on a detailed primary exposition by the framework’s originators. Future systematic reviews, preregistered longitudinal studies, and intervention research could strengthen the model’s empirical basis.

ISAF-DCD should therefore be treated as a theoretically grounded heuristic and a source of testable hypotheses, rather than as a definitive account of DCD psychopathology. Its value will depend on whether future research can distinguish shared vulnerability from experiential mediation, identify reciprocal pathways, clarify developmental differences, and determine which interventions promote positive adaptation in individuals with DCD and co-occurring neurodevelopmental conditions.

## Data Availability

The original contributions presented in the study are included in the article/supplementary material, further inquiries can be directed to the corresponding author.

## References

[ref1] Abregú-Crespo R. Garriz-Luis A. Ayora M. Martín-Martínez N. Cavone V. Carrasco M. Á. . (2024). School bullying in children and adolescents with neurodevelopmental and psychiatric conditions: a systematic review and meta-analysis. Lancet Child Adolesc. Health 8, 122–134. doi: 10.1016/S2352-4642(23)00289-4, 38109913

[ref2] American Psychiatric Association (2022). Diagnostic and Statistical Manual of Mental Disorders. 5th Edn. Washington, DC: American Psychiatric Association.

[ref3] Antolini G. Colizzi M. (2023). Where do neurodevelopmental disorders go? Casting the eye away from childhood towards adulthood. Healthcare 11:1015. doi: 10.3390/healthcare11071015, 37046942 PMC10094062

[ref4] Bandura A. (1997). Self-Efficacy: The Exercise of Control. New York: W. H. Freeman/Times Books/Henry Holt & Co.

[ref5] Black M. H. Helander J. Segers J. Ingard C. Bervoets J. de Puget V. G. . (2024). Resilience in the face of neurodivergence: a scoping review of resilience and factors promoting positive outcomes. Clin. Psychol. Rev. 113:102487. doi: 10.1016/j.cpr.2024.102487, 39178757

[ref6] Blank R. Barnett A. L. Cairney J. Green D. Kirby A. Polatajko H. . (2019). International clinical practice recommendations on the definition, diagnosis, assessment, intervention, and psychosocial aspects of developmental coordination disorder. Dev. Med. Child Neurol. 61, 242–285. doi: 10.1111/dmcn.14132, 30671947 PMC6850610

[ref7] Blasco P. Saxton S. Gullion L. M. Oo T. Z. Amukune S. Józsa K. (2023). Assessment of mastery motivation and neurodevelopment of young children at high risk for developmental delays. J. Intelligence 11:115. doi: 10.3390/jintelligence11060115, 37367517 PMC10299690

[ref8] Broletti M. C. Efthymiou C. Murray A. L. McDougal E. Rhodes S. M. (2024). Investigating the mediating role of executive function in the relationship between ADHD and DCD symptoms and depression in adults. J. Autism Dev. Disord. 54, 4684–4696. doi: 10.1007/s10803-023-06148-7, 37966535 PMC11549189

[ref9] Burton B. K. Krantz M. F. Skovgaard L. T. Brandt J. M. Gregersen M. Søndergaard A. . (2023). Impaired motor development in children with familial high risk of schizophrenia or bipolar disorder and the association with psychotic experiences: a 4-year Danish observational follow-up study. Lancet Psychiatry 10, 108–118. doi: 10.1016/S2215-0366(22)00401-5, 36610442

[ref10] Caçola P. (2016). Physical and mental health of children with developmental coordination disorder. Front. Public Health 4:224. doi: 10.3389/fpubh.2016.00224, 27822464 PMC5075567

[ref11] Cairney J. (2015). Developmental Coordination Disorder and its Consequences. Toronto, Canada: University of Toronto Press.

[ref12] Cairney J. (2018). Quality of life and psychosocial functioning in adolescents with developmental coordination disorder and attention-deficit-hyperactivity disorder. Dev. Med. Child Neurol. 60:643. doi: 10.1111/dmcn.13796, 29727010

[ref13] Cairney J. Rigoli D. Piek J. (2013). Developmental coordination disorder and internalizing problems in children: the environmental stress hypothesis elaborated. Dev. Rev. 33, 224–238. doi: 10.1016/j.dr.2013.07.002

[ref14] Cairney J. Veldhuizen S. Szatmari P. (2010). Motor coordination and emotional-behavioral problems in children: a test of the environmental stress hypothesis. J. Pediatr. 156, 924–927. doi: 10.1016/j.jpeds.2010.01.023 20520549

[ref15] Carragher N. Krueger R. F. Eaton N. R. Slade T. (2015). Disorders without borders: current and future directions in the meta-structure of mental disorders. Soc. Psychiatry Psychiatr. Epidemiol. 50, 339–350. doi: 10.1007/s00127-014-1004-z, 25557024

[ref16] Cavalcante J. L. Draghi T. T. G. (2021). Developmental coordination disorder and the COVID-19 pandemic: some considerations. Motriz Rev. Educ. Fis. 27:e10200226. doi: 10.1590/S1980-657420210000226

[ref17] Cavalcante Neto J. L. Draghi T. T. G. Santos I. W. P. D. Brito R. D. S. Silva L. S. O. Lima U. D. S. (2024). Physical fitness in children with developmental coordination disorder: a systematic review. Phys. Occup. Ther. Pediatr. 44, 626–655. doi: 10.1080/01942638.2024.2327354, 38587180

[ref18] Cavalcante-Neto J. L. Bourke M. Silva J. M. C. Cairney J. (2026). Emotional outcomes are poorer in adults with developmental coordination disorder: a systematic review and meta-analyses. J. Psychosom. Res. 202:112537. doi: 10.1016/j.jpsychores.2026.112537, 41529439

[ref19] Cleaton M. A. M. Lorgelly P. K. Kirby A. (2019). Developmental coordination disorder: the impact on the family. Qual. Life Res. 28, 925–934. doi: 10.1007/s11136-018-2075-1, 30536221

[ref20] Curșeu P. L. van Rijswijk J. (2026). Different minds, shared struggles: personality and self-beliefs across neurodivergent conditions. Acta Psychol. 267:106955. doi: 10.1016/j.actpsy.2026.106955, 42161138

[ref21] Dahl R. E. Armstrong-Carter E. van den Bos W. (2024). Wanting to matter and learning to care: a neurodevelopmental window of opportunity for (pro)social learning? Dev. Cogn. Neurosci. 69:101430. doi: 10.1016/j.dcn.2024.101430, 39151254 PMC11377138

[ref22] De Melo K. De Oliveira J. Niechwiej-Szwedo E. Schott N. Vallis L. A. (2026). Pediatric developmental coordination disorder screening and assessment: emerging roles of technology. Clin. Pediatr. 24:99228261476200. doi: 10.1177/00099228261476200, 42638393

[ref23] De Roubaix A. Warlop G. Van Dyck D. Van Crombrugge D. Van den Abbeele S. Licari M. . (2025). Understanding the impact of developmental coordination disorder on Belgian children and families: a national survey study. PLoS One 20:e0320311. doi: 10.1371/journal.pone.0320311, 40261859 PMC12013903

[ref24] Demartini B. Nisticò V. Edwards M. J. Gambini O. Priori A. (2020). The pathophysiology of functional movement disorders. Neurosci. Biobehav. Rev. 120, 387–400. doi: 10.1016/j.neubiorev.2020.10.019, 33159917

[ref25] Douka I. Ruitenberg M. F. L. Weischedel K. Phouthavongsay C. Weisenbach S. L. van der Geest J. N. . (2025). Cognitive and motor disturbances in depression: insights from comprehensive behavioral assessments. Front. Psych. 16:1624776. doi: 10.3389/fpsyt.2025.1624776, 40800628 PMC12340722

[ref26] Draghi T. T. G. Cavalcante Neto J. L. Rohr L. A. Jelsma L. D. Tudella E. (2020). Symptoms of anxiety and depression in children with developmental coordination disorder: a systematic review. J. Pediatr. 96, 8–19. doi: 10.1016/j.jped.2019.03.002, 31029680 PMC9432331

[ref27] Draghi T. T. G. Cavalcante Neto J. L. Tudella E. (2021). Symptoms of anxiety and depression in schoolchildren with and without developmental coordination disorder. J. Health Psychol. 26, 1519–1527. doi: 10.1177/1359105319878253, 31556324

[ref28] Dreiskämper D. Tietjens M. Schott N. (2022). The physical self-concept across childhood: measurement development and meaning for physical activity. Psychol. Sport Exerc. 61, 102187–102114. doi: 10.1016/j.psychsport.2022.102187, 42574925

[ref29] Dunford C. Missiuna C. Street E. Sibert J. (2005). Children’s perceptions of the impact of developmental coordination disorder on activities of daily living. Br. J. Occup. Ther. 68, 207–214. doi: 10.1177/030802260506800504

[ref30] Engel-Yeger B. (2025). Developmental coordination disorder: emotional and cognitive implications on adults’ quality of life. Can J Occup Ther. 92:323–336. doi: 10.1177/00084174251333392 40255132

[ref31] Engel-Yeger B. Engel A. (2023). Emotional distress and quality of life among adults with developmental coordination disorder during COVID-19. Br. J. Occup. Ther. 86, 130–138. doi: 10.1177/03080226221126892, 38603414 PMC9554548

[ref32] Erskine N. Barratt J. Cairney J. (2024). Paediatric motor difficulties and internalising problems: an integrative review on the environmental stress hypothesis. Front. Pediatr. 12:1320338. doi: 10.3389/fped.2024.1320338, 39156018 PMC11327034

[ref33] Fogel Y. Stuart N. Joyce T. Barnett A. L. (2023). Relationships between motor skills and executive functions in developmental coordination disorder (DCD): a systematic review. Scand. J. Occup. Ther. 30, 344–356. doi: 10.1080/11038128.2021.2019306, 34962855

[ref34] Fortnum K. Erskine N. Cairney J. Kwan M. Y. W. Bourke M. (2026). Co-occurring psychiatric disorders and symptomatology among children and adolescents with neurodevelopmental disorders: an umbrella review with individual study meta-analysis. Child Adolesc Ment Health. doi: 10.1111/camh.70093 42047642

[ref35] Fulun L. Yanan L. Bing G. Qian L. Aishu L. Xia L. . (2025). Hypothalamic-pituitary-adrenal axis dysfunction in children with ADHD: a systematic review and meta-analysis. Psychoneuroendocrinology 181:107605. doi: 10.1016/j.psyneuen.2025.107605, 40974807

[ref36] Gambra L. Cortese S. Lizoain P. Romero D. R. Paiva U. Gándara C. . (2024). Excessive body weight in developmental coordination disorder: a systematic review and meta-analysis. Neurosci. Biobehav. Rev. 164:105806. doi: 10.1016/j.neubiorev.2024.105806, 38986892

[ref37] Godoi-Jacomassi D. Smits-Engelsman B. Draghi T. T. G. Tudella E. Jelsma D. (2025). Underlying neurophysiological and biomechanical mechanisms related to postural control of children with developmental coordination disorder differ from those in typically developing children. Res. Dev. Disabil. 158:104939. doi: 10.1016/j.ridd.2025.104939, 39913959

[ref38] Gras D. Ploix Maes E. Doulazmi M. Huron C. Galléa C. Boespflug Tanguy O. . (2023). Developmental coordination disorder subtypes in children: an unsupervised clustering. Dev. Med. Child Neurol. 65, 1332–1342. doi: 10.1111/dmcn.15563, 36883642

[ref39] Green D. Dewey D. (2026). Movement function and performance in children with DCD or ASD. Curr. Dev. Disord. Rep. 13:3. doi: 10.1007/s40474-026-00347-4

[ref40] Hammen C. (1991). Generation of stress in the course of unipolar depression. J. Abnorm. Psychol. 100, 555–561. doi: 10.1037/0021-843X.100.4.555, 1757669

[ref41] Harris S. Rathbone C. J. Wilmut K. (2024). The role of anxiety and self-efficacy in movement. Hum. Mov. Sci. 98:103306. doi: 10.1016/j.humov.2024.103306, 39591810

[ref42] Harris S. Rathbone C. J. Wilmut K. (2025). Does how I feel change how I move? The influence of anxiety, self-efficacy and resilience on movement in adults with developmental coordination disorder. Res. Dev. Disabil. 158:104927. doi: 10.1016/j.ridd.2025.104927, 39892033

[ref43] Harrowell I. Hollén L. I. Lingam R. Emond A. M. (2017). Mental health outcomes of developmental coordination disorder in late adolescence. Dev. Med. Child Neurol. 59, 973–979. doi: 10.1111/dmcn.13469, 28512766 PMC5573907

[ref44] Healy C. Brannigan R. Dooley N. Staines L. Keeley H. Whelan R. . (2022). Person-centered trajectories of psychopathology from early childhood to late adolescence. JAMA Netw. Open 5:e229601. doi: 10.1001/jamanetworkopen.2022.9601, 35536581 PMC9092205

[ref45] Hill E. L. Brown D. (2013). Mood impairments in adults previously diagnosed with developmental coordination disorder. J. Ment. Health 22, 334–340. doi: 10.3109/09638237.2012.745187, 23323694

[ref46] Hobfoll S. E. (1989). Conservation of resources: a new attempt at conceptualizing stress. Am. Psychol. 44, 513–524. doi: 10.1037/0003-066X.44.3.513, 2648906

[ref47] Idan O. Braun-Lewensohn O. Lindström B. Margalit M. (2022). Salutogenesis: sense of coherence in childhood and families. In Mittelmark M.B. Bauer G.F. Vaandrager L. Pelikan J.M. Sagy S. Eriksson M. . The Handbook of Salutogenesis. (2nd. Cham: Springer. pp. 123–138). 36122025

[ref48] Jacobson N. C. Newman M. G. (2014). Avoidance mediates the relationship between anxiety and depression over a decade later. J. Anxiety Disord. 28, 437–445. doi: 10.1016/j.janxdis.2014.03.007, 24858656 PMC4957550

[ref49] James M. E. Graham J. D. Chirico D. King-Dowling S. Cairney J. (2022). Investigating the mediating role of internalizing and externalizing problems on physical fitness in children at risk for developmental coordination disorder. Appl. Physiol. Nutr. Metab. 47, 575–581. doi: 10.1139/apnm-2021-0369 35167349

[ref50] Järvinen I. Launes J. Lipsanen J. Lehto E. Schiavone N. Virta M. . (2024). Motor difficulties from childhood to midlife: a 40-year cohort study. Res. Dev. Disabil. 146:104670. doi: 10.1016/j.ridd.2024.104670, 38237252

[ref51] Jijon A. M. Leonard H. C. (2020). Parenting stress in parents of children with developmental coordination disorder. Res. Dev. Disabil. 104:103695. doi: 10.1016/j.ridd.2020.103695, 32474232

[ref52] Ketcheson L. R. Pitchford E. A. Wentz C. F. (2021). The relationship between developmental coordination disorder and concurrent deficits in social communication and repetitive behaviors among children with autism spectrum disorder. Autism Res. 14, 804–816. doi: 10.1002/aur.2469, 33421296

[ref53] Klein E. S. Licari M. Barbic S. Zwicker J. G. (2024). Diagnostic services for developmental coordination disorder: gaps and opportunities identified by parents. Child Care Health Dev. 50:e13230. doi: 10.1111/cch.13230, 38265129

[ref54] Lachambre C. Proteau-Lemieux M. Lepage J. F. Bussières E. L. Lippé S. (2021). Attentional and executive functions in children and adolescents with developmental coordination disorder and the influence of comorbid disorders: a systematic review of the literature. PLoS One 16:e0252043. doi: 10.1371/journal.pone.0252043, 34086687 PMC8177544

[ref55] Lazarus R. S. Folkman S. (1984). Stress, Appraisal, and Coping. New York, NY: Springer Publishing Company.

[ref56] Li Y. C. Graham J. D. Cairney J. (2018). Moderating effects of physical activity and global self-worth on internalizing problems in school-aged children with developmental coordination disorder. Front. Psychol. 9:1740. doi: 10.3389/fpsyg.2018.01740, 30283385 PMC6157544

[ref57] Li J. Herold F. Chen Y. Zhang Z. Werneck A. O. Ji T. . (2025). Longitudinal associations between physical activity intensity and mental health problems in overweight/obese youth. Appl. Psychol. Health Well Being 17:e70071. doi: 10.1111/aphw.70071, 40831329

[ref58] Li H. Ke X. Huang D. Xu X. Tian H. Gao J. (2024). The prevalence of developmental coordination disorder in children: a systematic review and meta-analysis. Front. Pediatr. 12:1387406. doi: 10.3389/fped.2024.1387406, 39391054 PMC11464289

[ref59] Luiz A. C. M. Tsutsumi M. M. A. (2026). The ontology of mental health disorders: embracing the diathesis-stress model. Einstein (São Paulo) 24:eAE1796. doi: 10.31744/einstein_journal/2026AE1796, 41538684 PMC13004543

[ref60] Lust J. M. Steenbergen B. Diepstraten J. A. E. M. Wilson P. H. Schoemaker M. M. Poelma M. J. (2022). The subtypes of developmental coordination disorder. Dev. Med. Child Neurol. 64, 1366–1374. doi: 10.1111/dmcn.15260, 35578393 PMC9790450

[ref61] Mancini V. O. Licari M. K. Alvares G. A. McQueen M. C. McIntyre S. Reynolds J. E. . (2024). Psychosocial well-being, parental concerns, and familial impact of children with developmental coordination disorder. Res. Dev. Disabil. 145:104659. doi: 10.1016/j.ridd.2023.104659, 38160588

[ref62] Martin N. C. Piek J. P. Hay D. (2006). DCD and ADHD: a genetic study of their shared aetiology. Hum. Mov. Sci. 25, 110–124. doi: 10.1016/j.humov.2005.10.006, 16442650

[ref63] Meachon E. J. Alpers G. W. (2023). How are you getting by? Coping in developmental coordination disorder versus attention-deficit/hyperactivity disorder. Br. J. Occup. Ther. 86, 42–52. doi: 10.1177/03080226221113505, 40337183 PMC12033471

[ref64] Meachon E. J. Melching H. Alpers G. W. (2024). The overlooked disorder: (un)awareness of developmental coordination disorder across clinical professions. Adv. Neurodev. Disord. 8, 253–261. doi: 10.1007/s41252-023-00334-5

[ref65] Meachon E. J. Schaider J. P. Alpers G. W. (2025). Motor skills in children with ADHD: overlap with developmental coordination disorder. BMC Psychol. 13:53. doi: 10.1186/s40359-024-02282-8, 39827182 PMC11742537

[ref66] Medeiros P. Cardoso F. L. Cordeiro P. C. Paiva Vieira E. Silva W. R. D. Tamplain P. (2023). Self-esteem, self-efficacy, and social support mediate the relationship between motor proficiency and internalizing problems in adults: exploring the environmental stress hypothesis in adulthood. Hum. Mov. Sci. 88:103072. doi: 10.1016/j.humov.2023.103072, 36867976

[ref67] Medeiros P. Cardoso F. L. Silva W. R. D. Zequinão M. A. Tamplain P. (2022). Externalizing problems mediate the relationship between motor proficiency and internalizing problems in children: an extension of the environmental stress hypothesis. Hum. Mov. Sci. 81:102916. doi: 10.1016/j.humov.2021.102916 34953291

[ref68] Micai M. Fatta L. M. Gila L. Caruso A. Salvitti T. Fulceri F. . (2023). Prevalence of co-occurring conditions in children and adults with autism spectrum disorder: a systematic review and meta-analysis. Neurosci. Biobehav. Rev. 155:105436. doi: 10.1016/j.neubiorev.2023.105436 37913872

[ref69] Michelini G. Carlisi C. O. Eaton N. R. Elison J. T. Haltigan J. D. Kotov R. . (2024). Where do neurodevelopmental conditions fit in transdiagnostic psychiatric frameworks? Incorporating a new neurodevelopmental spectrum. World Psychiatry 23, 333–357. doi: 10.1002/wps.21225, 39279404 PMC11403200

[ref70] Morgan G. A. Harmon R. J. Maslin-Cole C. (1990). Mastery motivation: definition and measurement. Early Educ. Dev. 1, 317–339. doi: 10.1207/s15566935eed0104_7

[ref71] Mountford H. S. Hill A. Barnett A. L. Newbury D. F. (2021). Genome-wide association study of motor coordination. Front. Hum. Neurosci. 15:669902. doi: 10.3389/fnhum.2021.669902, 34177493 PMC8219980

[ref72] Murray R. Staniforth C. E. Shawak A. Eddy L. H. (2025). Developmental coordination disorder: “it’s not on people’s radars… they’re not interested.”. PLoS One 20:e0337001. doi: 10.1371/journal.pone.0337001, 41252385 PMC12626263

[ref73] Nobusako S. Sakai A. Tsujimoto T. Shuto T. Nishi Y. Asano D. . (2018). Deficits in visuo-motor temporal integration impacts manual dexterity in probable developmental coordination disorder. Front. Neurol. 9:114. doi: 10.3389/fneur.2018.00114, 29556211 PMC5844924

[ref74] Omer S. Jijon A. M. Leonard H. C. (2019). Research review: Internalising symptoms in developmental coordination disorder: a systematic review and meta-analysis. J. Child Psychol. Psychiatry 60, 606–621. doi: 10.1111/jcpp.13001, 30485419 PMC7379561

[ref75] Omer S. Leonard H. C. (2021). Internalising symptoms in developmental coordination disorder: the indirect effect of everyday executive function. Res. Dev. Disabil. 109:103831. doi: 10.1016/j.ridd.2020.103831, 33360963

[ref77] Pelletier-Baldelli A. Sheridan M. A. Glier S. Rodriguez-Thompson A. Gates K. M. Martin S. . (2022). Social goals in girls transitioning to adolescence: associations with psychopathology and brain network connectivity. Soc. Cogn. Affect. Neurosci. 18:nsac058. doi: 10.1093/scan/nsac058, 36287067 PMC9949572

[ref78] Piek J. P. Rigoli D. Pearsall-Jones J. G. Martin N. C. Hay D. A. Bennett K. S. . (2007). Depressive symptomatology in child and adolescent twins with attention-deficit hyperactivity disorder and/or developmental coordination disorder. Twin Res. Hum. Genet. 10, 587–596. doi: 10.1375/twin.10.4.587 17708700

[ref79] Poletti M. Raballo A. (2022). (developmental) motor signs: Reconceptualizing a potential transdiagnostic marker of psychopathological vulnerability. Schizophr. Bull. 48, 763–765. doi: 10.1093/schbul/sbac026, 35265980 PMC9212093

[ref80] Pranjić M. Rahman N. Kamenetskiy A. Mulligan K. Pihl S. Arnett A. B. (2023). A systematic review of behavioral and neurobiological profiles associated with coexisting attention-deficit/hyperactivity disorder and developmental coordination disorder. Neurosci. Biobehav. Rev. 153:105389. doi: 10.1016/j.neubiorev.2023.105389, 37704094 PMC12042734

[ref81] Pruessner M. Cullen A. E. Aas M. Walker E. F. (2017). The neural diathesis-stress model of schizophrenia revisited: an update on recent findings considering illness stage and neurobiological and methodological complexities. Neurosci. Biobehav. Rev. 73, 191–218. doi: 10.1016/j.neubiorev.2016.12.013 27993603

[ref82] Purcell C. Schott N. Rapos V. Zwicker J. G. Wilmut K. (2023). Understanding factors that influence physical activity behavior in people with developmental coordination disorder (DCD): a mixed-methods convergent integrated systematic review. Front. Hum. Neurosci. 17:1274510. doi: 10.3389/fnhum.2023.1274510, 38152480 PMC10751368

[ref83] Rasmussen P. Gillberg C. (2000). Natural outcome of ADHD with developmental coordination disorder at age 22 years: a controlled, longitudinal, community-based study. J. Am. Acad. Child Adolesc. Psychiatry 39, 1424–1431. doi: 10.1097/00004583-200011000-00017, 11068898

[ref84] Rathbone C. J. Wilmut K. (2025). “I am dyspraxic”: self-concept and well-being in adults with developmental coordination disorder. Adv. Neurodev. Disord. 9:637–654. doi: 10.1007/s41252-025-00436-2

[ref85] Rodriguez M. C. Wade T. J. Veldhuizen S. Missiuna C. Timmons B. W. Cairney J. (2019). Emotional and behavioral problems in 4- and 5-year old children with and without motor delays. Front. Pediatr. 7:474. doi: 10.3389/fped.2019.00474, 31803697 PMC6877720

[ref86] Santee A. C. Rnic K. Chang K. K. Chen R. X. Hoffmeister J. A. Liu H. . (2023). Risk and protective factors for stress generation: a meta-analytic review. Clin. Psychol. Rev. 103:102299. doi: 10.1016/j.cpr.2023.102299, 37307790

[ref87] Sauer C. Wells M. B. Johansen K. (2026). Self-reported motor difficulties in adults with childhood developmental coordination disorder: a 25-year follow-up. Disabil. Rehabil. 48, 4856–4869. doi: 10.1080/09638288.2026.2616348, 41566665

[ref88] Scheel Rasmussen I. Wilson P. Overbeck G. Ekstrøm C. T. Olsen E. M. Strandberg-Larsen K. (2026). Mapping trajectories of child and adolescent psychopathology: ascertainment of mental health needs in a general population. Epidemiol. Psychiatr. Sci. 35:e20. doi: 10.1017/S2045796026100596, 41913473 PMC13122543

[ref89] Schneider J. L. West K. L. (2025). A matter of time: developmental cascades for developmental science. Infant Behav. Dev. 80:102078. doi: 10.1016/j.infbeh.2025.102078, 40489911

[ref90] Schott N. (2019). Dual-task performance in developmental coordination disorder (DCD): understanding trade-offs and their implications for training. Curr. Dev. Disord. Rep. 6, 87–101. doi: 10.1007/s40474-019-00163-z

[ref91] Scrocco A. Ferragina F. Bianucci C. Di Lieto M. C. Sgandurra G. (2026). The mirror neuron system in typically and atypically developing children: evolution, dysfunction and rehabilitative purpose. A systematic review. Neurosci. Biobehav. Rev. 189:106882. doi: 10.1016/j.neubiorev.2026.106882, 42480680

[ref92] Sisk L. M. Gee D. G. (2022). Stress and adolescence: vulnerability and opportunity during a sensitive window of development. Curr. Opin. Psychol. 44, 286–292. doi: 10.1016/j.copsyc.2021.10.005, 34818623 PMC9007828

[ref93] Sisk L. M. Keding T. J. Drew A. Ma E. Shafiei G. Cieslak M. . (2026). Corticolimbic Structure-Function Coupling Is Sensitive to Childhood Adversity and Buffers Adversity-Related Symptoms during Development. The Journal of neuroscience: the official journal of the Society for Neuroscience. 46:e2327252026. doi: 10.1523/JNEUROSCI.2327-25.2026 PMC13583330 42613185

[ref94] Smith K. E. Pollak S. D. (2020). Rethinking concepts and categories for understanding the neurodevelopmental effects of childhood adversity. Perspect. Psychol. Sci. 16, 67–93. doi: 10.1177/1745691620920725, 32668190 PMC7809338

[ref95] Southon C. (2022). The relationship between executive function, neurodevelopmental disorder traits, and academic achievement in university students. Front. Psychol. 13:958013. doi: 10.3389/fpsyg.2022.958013, 36118426 PMC9478894

[ref96] Steenbergen B. Valtr L. Dunford C. Prunty M. Bekhuis H. Temlali T. Y. . (2024). Awareness about developmental coordination disorder. Front. Public Health 12:1345257. doi: 10.3389/fpubh.2024.1345257, 38362216 PMC10867205

[ref97] Stults-Kolehmainen M. A. Sinha R. (2014). The effects of stress on physical activity and exercise. Sports Med. 44, 81–121. doi: 10.1007/s40279-013-0090-5, 24030837 PMC3894304

[ref98] Subara-Zukic E. Cole M. H. McGuckian T. B. Steenbergen B. Green D. Smits-Engelsman B. C. . (2022). Behavioral and neuroimaging research on developmental coordination disorder (DCD): a combined systematic review and meta-analysis of recent findings. Front. Psychol. 13:809455. doi: 10.3389/fpsyg.2022.809455, 35153960 PMC8829815

[ref99] Tal-Saban M. Yochman A. Weintraub N. Magen H. (2025). The sensory profile of students with probable developmental coordination disorder (DCD). Sci. Rep. 15:33270. doi: 10.1038/s41598-025-18361-8, 41006524 PMC12475141

[ref100] Tal-Saban M. Zarka S. Grotto I. Ornoy A. Parush S. (2012). The functional profile of young adults with suspected developmental coordination disorder (DCD). Res. Dev. Disabil. 33, 2193–2202. doi: 10.1016/j.ridd.2012.06.005, 22789703

[ref101] Van Dyck D. Baijot S. Aeby A. De Tiège X. Deconinck N. (2022). Cognitive, perceptual, and motor profiles of school-aged children with developmental coordination disorder. Front. Psychol. 13:860766. doi: 10.3389/fpsyg.2022.860766, 35992485 PMC9381813

[ref102] Verbecque E. Lust J. M. Smits-Engelsman B. (2026). Subtypes in developmental coordination disorder: what do we know so far? A systematic review. Eur. J. Pediatr. 185:510. doi: 10.1007/s00431-026-07180-2, 42322424

[ref103] Verlinden S. Wijngaert P. D. den Eynde J. V. (2023). Developmental coordination disorder in adults: a case series of a condition that is underdiagnosed by adult psychiatrists. Psychiatry Res. Case Rep. 2:100148. doi: 10.1016/j.psycr.2023.100148

[ref104] Waszczuk M. A. Leonard H. C. Hill E. L. Rowe R. Gregory A. M. (2016). Coordination difficulty and internalizing symptoms in adults: a twin/sibling study. Psychiatry Res. Neuroimaging 239, 1–8. doi: 10.1016/j.pscychresns.2016.02.044, 27137955

[ref105] Weber M. D. Draghi T. T. G. Maia A. C. S. Cairney J. Cavalcante-Neto J. L. Tudella E. (2025). Characteristics of sensory processing changes in children with developmental coordination disorder: a systematic review. Res. Dev. Disabil. 157:104917. doi: 10.1016/j.ridd.2025.104917, 39793213

[ref106] Weber M. D. Draghi T. T. G. Rohr L. A. Cavalcante Neto J. L. Tudella E. (2023). Health-related quality of life in children with developmental coordination disorder: a systematic review. Health Qual. Life Outcomes 21:62. doi: 10.1186/s12955-023-02146-6, 37386629 PMC10308763

[ref107] Yao B. Rolfs M. Slate R. Roberts D. Fattal J. Achtyes E. D. . (2024). Abnormal oculomotor corollary discharge signaling as a trans-diagnostic mechanism of psychosis. Schizophr. Bull. 50, 631–641. doi: 10.1093/schbul/sbad180, 38245499 PMC11059795

[ref108] Zaguri-Vittenberg S. Weintraub N. Tal-Saban M. (2026). Developmental coordination disorder and executive function deficits: implications for emotional, mental, and overall well-being. Braz. J. Phys. Ther. 30:101537. doi: 10.1016/j.bjpt.2025.101537, 41016236 PMC12510207

[ref109] Zuckerman M. (1999). Diathesis-stress models. In Zuckerman M., Vulnerability to Psychopathology: a Biosocial model. Washington, DC: American Psychological Association. (pp. 3–23).

